# Supplementary material for: Measurement invariance of the distress tolerance scale among university students with and without a history of non-suicidal self-injury
Source: PeerJ. 2021 Mar 15;9:e10915. doi: 10.7717/peerj.10915 (PMC7971082; doi:10.7717/peerj.10915)
Supplement: Supplemental Information 1 [file peerj-09-10915-s001.docx]

| Supplementary Table S1  *Distress Tolerance Scale item means and standard deviations for the total sample, individuals with a history of NSSI, and individuals without a history of NSSI* | | | |
| --- | --- | --- | --- |
|  | Total Sample (M, SD)  N = 531 | NSSI history (M, SD)  N = 215 | No NSSI history (M, SD)  N = 316 |
| Item 1 | 2.87 (1.15) | 2.46 (1.07) | 3.15 (1.11) |
| Item 2 | 2.63 (1.16) | 2.14 (1.03) | 2.96 (1.13) |
| Item 3 | 3.02 (1.22) | 2.53 (1.20) | 3.35 (1.12) |
| Item 4 | 3.04 (1.34) | 2.42 (1.27) | 3.46 (1.23) |
| Item 5 | 2.94 (1.30) | 2.60 (1.31) | 3.16 (1.24) |
| Item 6 | 3.24 (1.16) | 2.76 (1.13) | 3.56 (1.07) |
| Item 7 | 3.26 (1.78) | 2.84 (1.18) | 3.54 (1.08) |
| Item 8 | 2.83 (1.11) | 2.68 (1.19) | 2.92 (1.05) |
| Item 9 | 2.87 (1.20) | 2.33 (1.12) | 3.23 (1.11) |
| Item 10 | 3.01 (1.22) | 2.56 (1.16) | 3.31 (1.16) |
| Item 11 | 3.02 (1.35) | 2.47 (1.29) | 3.40 (1.26) |
| Item 12 | 3.13 (1.33) | 2.58 (1.29) | 3.51 (1.22) |
| Item 13 | 2.95 (1.16) | 2.68 (1.17) | 3.14 (1.12) |
| Item 14 | 2.91 (1.09) | 2.73 (1.11) | 3.03 (1.07) |
| Item 15 | 2.76 (1.21) | 2.20 (1.10) | 3.13 (1.14) |
