# Supplementary material for: Measurement invariance of the distress tolerance scale among university students with and without a history of non-suicidal self-injury
Source: PeerJ. 2021 Mar 15;9:e10915. doi: 10.7717/peerj.10915 (PMC7971082; doi:10.7717/peerj.10915)
Supplement: Supplemental Information 2 [file peerj-09-10915-s002.docx]

| Table S2  *Measurement invariance of the first-order four factor Distress Tolerance Scale* *between males and females* | | | | | | | | | | | | | |
| --- | --- | --- | --- | --- | --- | --- | --- | --- | --- | --- | --- | --- | --- |
|  | χ^2^ | *df* | Δ χ^2^ (Δ df) | *p* Δ χ^2^ | NCI | CFI | RMSEA | SRMR | Model Comparison | ΔNCI | ΔCFI | ΔRMSEA | ΔSRMR |
| Model 1: Configural invariance | 295.89 | 164 | - | - | 0.8830 | 0.949 | 0.055 | 0.050 |  | - | - | - | - |
| Model 2: Full metric invariance | 310.534 | 175 | 14.65 (11) | .199 | 0.8800 | 0.948 | 0.054 | 0.060 | M1-M2 | 0.0030 | 0.001 | 0.001 | 0.010 |
| Model 3: Full scalar invariance | 325.212 | 186 | 14.68 (11) | .198 | 0.8769 | 0.947 | 0.053 | 0.063 | M2-M3 | 0.0031 | 0.001 | 0.001 | 0.003 |
| Model 4: Full residual error invariance | 329.983 | 201 | 4.77 (15) | .994 | 0.8854 | 0.951 | 0.049 | 0.062 | M3-M4 | 0.0085 | 0.004 | .004 | 0.001 |
