# Supplementary material for: Measurement invariance of the distress tolerance scale among university students with and without a history of non-suicidal self-injury
Source: PeerJ. 2021 Mar 15;9:e10915. doi: 10.7717/peerj.10915 (PMC7971082; doi:10.7717/peerj.10915)
Supplement: Supplemental Information 4 [file peerj-09-10915-s004.docx]

| **Codebook for Measurement Invariance DTS dataset** | |
| --- | --- |
| **Construct/Measure** | **Coding** |
| Gender | Male: 1  Female: 2 |
| History of NSSI (“Have you ever engaged in non-suicidal self-injury?”) | No: 0  Yes: 1 |
| Frequency of NSSI (“How many times have you self-injured in the last year?”) | 0: None  1: Once  2: Twice  3: Three times  4: Four times  5: Five or more times |
| Main form of self-injury | 1: Cutting  2: Biting  3: Burning  4: Carving  5: Pinching  6: Pulling hair  7: Severe scratching  8: Banging or hitting yourself  9: Interfering with wound healing  10: Rubbing skin against rough surface  11: Sticking yourself with needles  12: Swallowing dangerous substances  13: Other |
| DTS1-15 (DTS item 1 through DTS item 15) | Response scale: 1:Strongly agree; 2: Mildly agree; 3: Agree and disagree equally; 4: Mildly disagree; 5: Strongly disagree |
| DTS Tolerance subscale | (DTS_1 + DTS_3 + DTS_5) / 3 |
| DTS Appraisal subscale | (DTS_6_ReverseScored^a^ + DTS_7 + DTS_9 + DTS_10 + DTS_11 + DTS_12) / 6 |
| DTS Absorption subscale | (DTS_2 + DTS_4 + DTS_15) / 3 |
| DTS Regulation subscale | (DTS_8 + DTS_13 + DTS_14) / 3 |
| DTS Total | (DTS_Tolerance + DTS_Appraisal + DTS_Absorption + DTS_Regulation) / 4 |
| ^a^Item 6 on the DTS has already been reverse coded in the submitted dataset | |
